# Ectoparasite loads in sympatric urban populations of the northern white-breasted and the European hedgehog

**DOI:** 10.1007/s00436-015-4427-x

**Published:** 2015-03-29

**Authors:** Sylwia Dziemian, Bożena Sikora, Barbara Piłacińska, Jerzy Michalik, Rafał Zwolak

**Affiliations:** 1 Department of Systematic Zoology, Faculty of Biology, Adam Mickiewicz University, Umultowska 89, 61-614 Poznań, Poland; 2 Department of Animal Morphology, Faculty of Biology, Adam Mickiewicz University, Poznań, Poland

**Keywords:** Ectoparasites, *Erinaceus europaeus*, *Erinaceus roumanicus*, Fleas, Hedgehogs, Ticks

## Abstract

We investigated abundance and prevalence of ticks and fleas infesting urban populations of two species of hedgehogs: the northern white-breasted hedgehog (*Erinaceus roumanicus*) and the European hedgehog (*Erinaceus europaeus*). The hedgehogs were captured in the city of Poznań (western Poland) over the period of 4 years. Both species of hedgehogs were infested with the castor bean tick (*Ixodes ricinus*), the hedgehog tick (*Ixodes hexagonus*), and the hedgehog flea (*Archeopsylla erinacei*). The northern white-breasted hedgehog had higher loads of *I. ricinus* and *A. erinacei* than the European hedgehog. The abundance and prevalence of *I. hexagonus* were similar on both species of hosts. Co-infestation with the two species of ticks was more frequent on the northern white-breasted hedgehog than on the European hedgehog. Therefore, these two closely related species of hedgehogs differ in their importance as hosts of arthropod vectors of pathogens in urban areas and might play a different role in the dynamics of zoonotic diseases.

## Introduction

Ticks (Acari) and fleas (Siphonaptera) are among the most important hematophagous ectoparasites of terrestrial vertebrates and vectors of zoonotic disease. During their blood meals, they might transmit various pathogens such as viruses, bacteria, and protozoa that can infect animals and humans (Beugnet and Marié 2009; Sobrino et al. 2012). The increase in the incidence of flea- and tick-borne zoonotic diseases is facilitated by the development of extensive urban and suburban areas with gardens, parks, and other green spaces. This process provides suitable habitat for wild hosts of arthropod vectors, bringing humans and disease-transmitting arthropods in proximity and increasing the risk of infections (Patz et al. 2004; Beugnet and Marié 2009).

Hedgehogs (*Erinaceus* sp.) are a prominent example of wild animals that thrive in urban areas, are infested by ticks and fleas, and might facilitate infection of humans and their pets with zoonotic microorganisms (Riley and Chomel 2005; Hubert et al. 2011; Poel et al. 2015). Urban environment is often characterized by impoverished fauna. In particular, it lacks large vertebrates such as cervids, which serve as hosts for most of the adult ticks (Dautel and Kahl 1999). The insufficient availability of suitable hosts might often be a major factor restricting the occurrence of ticks in towns (Dautel and Kahl 1999). However, all developmental stages of many ticks feed on medium-sized mammals such as hedgehogs (Pfäffle et al. 2011; Dziemian et al. 2014). Moreover, hedgehogs are known to be very heavily infested with ticks and fleas (Gaglio et al. 2010; Földvári et al. 2011). Therefore, these mammals play an important role in the maintenance of zoonotic agents within urban areas (Dautel and Kahl 1999; Pfäffle et al. 2014).

Northern white-breasted hedgehogs (*Erinaceus roumanicus*) and European hedgehogs (*Erinaceus europaeus*) often co-occur in urban areas within their central European contact zone, which runs from central Scandinavia through western Poland, central and eastern Moravia, western and central Austria to western Slovenia, and Italy (Lapini 1999). Until 1967, the European hedgehog and the northern white-breasted hedgehog were not recognized as separate species, and even currently, very little is known about possible differences in their biology (Kral 1967). The research gaps include potential differences in ectoparasite infestation patterns between the two hedgehog species. Such studies are needed to evaluate the role of these mammals in the circulation of zoonotic diseases. The Europaean hedgehog is known to harbor several tick-borne pathogens such as *Borrelia burgdorferi* s.l. and *Anaplasma phagocytophilum*, and flea-borne pathogens like *Yersinia pseudotuberculosis* (Keymer et al. 1991; Gray et al. 1994; Gern et al. 1997; Skuballa et al. 2007; Skuballa et al. 2010). Data on the northern white-breasted hedgehog are scarce, but it is known to maintain TBE virus and, recently, it has been found to be a potential reservoir of Lyme disease spirochetes (Kožuch et al. 1967; Skuballa et al. 2012). Furthermore, urban populations of this hedgehog species are infected with *Candidatus* Neoehrlichia mikurensis and *A. phagocytophilum* (Földvári et al. 2014). The latter was also detected in castor bean ticks, *Ixodes ricinus*, removed from northern white-breasted hedgehogs in Romania and Hungary (Dumitrache et al. 2013; Földvári et al. 2014). Moreover, the hedgehog flea (*Archaeopsylla erinacei*), which infests both *E. europaeus* and *E. roumanicus*, was found to host *Ricketsia felis*, *Bartonella clarridgeiae*, and *Bartonella elizabethae* (Bitam et al. 2006; Gilles et al. 2009; Bitam et al. 2010; Hornok et al. 2014).

In general, hosts that are taxonomically related and ecologically similar are likely to share parasite species and thus have the potential of hosting similar pathogens (Bitam et al. 2010). However, we do not know if parasite loads of sympatric populations of the two hedgehog species differ. Research on ectoparasites of *E. roumanicus* and *E. europaeus* were conducted in areas of allopatry or focused on only one of these species (Gray et al. 1994; Reeve 1994; Pfäffle et al. 2009; Thamm et al. 2010; Földvári et al. 2011; Dziemian et al. 2014; Hajipour et al. 2014). Alternatively, inferences were based on ectoparasites collected from dead animals provided by wildlife rescue centers (Pfäffle et al. 2014). As a consequence, reliable comparisons of ectoparasite loads in *E. roumanicus* and *E. europaeus* are currently non-existent. Here, we describe patterns of ectoparasite infestation of both hedgehogs species co-occurring in the city of Poznan in western Poland. To our knowledge, this is the first study of ectoparasites of living hedgehogs in sympatric populations of *E. roumanicus* and *E. europaeus*.

## Materials and methods

Poznań (52° 17′ 34″ N, 16° 44′ 08″ E) is a city in western Poland with a population of 551,600 people and area of 262 km^2^. The hedgehogs were captured regularly within three residential areas: BON (60 ha), SOB (54 ha), and TYS (48 ha). They have also been caught haphazardly in a few areas and pooled into category “other” (OTH) or rescued from drainage ditches stretching along Poznan’s Fast Tram (PST) line. Hedgehog trapping was conducted from 2009 to 2012 throughout the period of hedgehog activity (March–November) by walking on established transects within the residential areas, starting at sunset and lasting until 23:00 to 2:00. Hedgehogs were located with flashlights and captured by hand. Checkups of drainage ditches along PST were performed two to three times per week during morning hours (8:00–10:00).

Captured hedgehogs were taken to the laboratory, were kept overnight in individual boxes (37 × 47 × 26 cm), and received commercial cat food and water ad libitum. In the morning, the animals were weighted, sexed, and placed on a white sheet of paper and visually examined for ectoparasites. The examination was usually performed by two cooperating researchers and lasted from 15 min to 2 h, depending on the degree of infestation and behavior of the animal. Some individuals did not unroll and were not included in the analyses. Hedgehogs were individually marked (with color-coded and numbered plastic tubes glued to spines) before releasing in the following evening in the exact place of capture (or, in case of PST, 100 m away from the ditches). In total, we examined 296 hedgehogs: 46 European hedgehogs (12 females and 34 males) and 250 northern white-breasted hedgehogs (120 females and 130 males), with 3 recaptures of the European hedgehog and 46 recaptures of the northern white-breasted hedgehog giving total sample size of 327. Catching and handling procedures of hedgehogs were approved by the appropriate Institutional Animal Care and Use Committees (permission no. DOPog-4201-03-158/03/al.). All fleas and feeding ticks found on hedgehogs were removed with tweezers and stored in 70 % alcohol. In addition, each box in which an animal was kept was examined for the presence of detached ticks. Ticks and fleas were counted and identified under a microscope using standard keys (Arthur 1963; Skuratowicz 1967; Siuda 1993). All ticks belonged to two species: the hedgehog tick, *Ixodes hexagonus*, and the castor bean tick, *I. ricinus*, and almost all fleas were identified as the hedgehog flea *A. erinacei.* Other fleas (six individuals of *Nosopsyllus fasciatus*, one *Ctenophthalmus agyrtes*, and one *Ctenophthalmus assimilis*) were not included in statistical analyses due to their rarity.

For each ectoparasite species, we estimated mean abundance (the number of a parasite species per host: Bush et al. 1997) and prevalence (the number of hosts infested with one or more individuals of a parasite: Bush et al. 1997) on both host species. We also compared proportions of *E. roumanicus* and *E. europeaus* hosts that were co-infested with both tick species. All analyses were conducted with generalized linear mixed models (GLMMs; Paterson and Lello 2003) implemented via the lme4 package (Bates et al. 2014) in R (R Core Team 2013). In the analysis of parasite abundance, we used Poisson family error terms and a log link function. In the analysis of prevalence and tick co-infestation, we used binomial family and logit link function. Predictor variables included fixed effects of host species (*E. roumanicus* vs. *E. europeaus*), host sex (male vs. female hedgehogs), and season (grouped as spring, April–May; summer, June–August; and fall, September–November). Additionally, in the analyses of tick abundance and prevalence, we controlled for tick stage (female, nymph, or larvae: entered as a fixed effect). The initial models included all possible two-way interactions among the fixed effects. To simplify the initial models, we eliminated in a stepwise fashion all interactions with *P* > 0.10.

Random effects included individual hedgehog, year, and study site. In the analyses of prevalence and abundance, random effects also included a unique identifier for each observation, which creates over-dispersed Poisson and binomial models (Zwolak et al. 2013). In the analysis of tick abundance, the effect of tick stage was partly correlated over individual host. However, we did not use this structure in the analyses of prevalence and co-infestation because it did not improve the fit of the models (evaluated with Akaike’s information criterion: Burnham and Anderson 2002).

## Results

### *Ixodes ricinus*

**Fig. 1:**
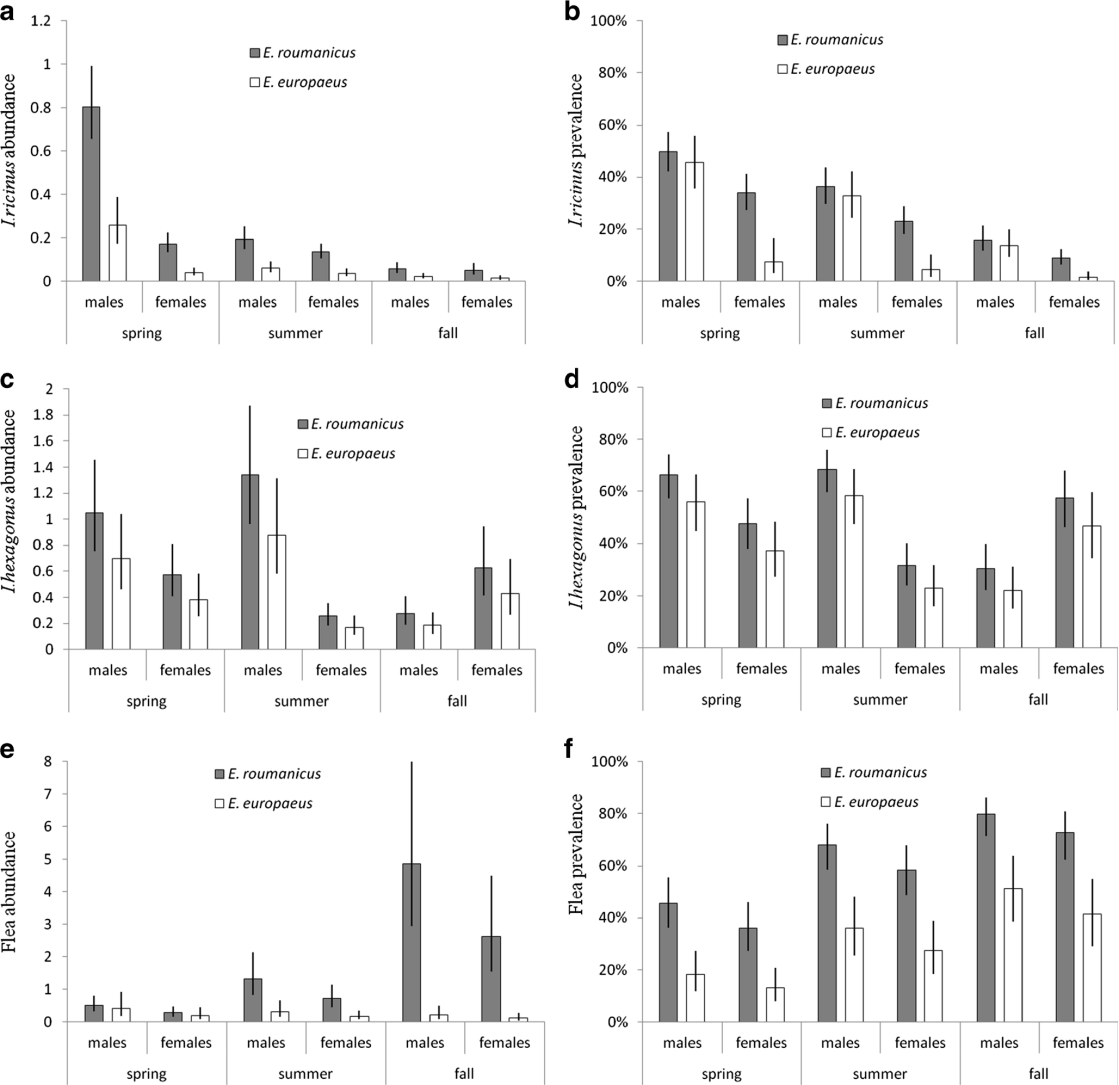
Abundance and prevalence of ectoparasites infesting the northern white-breasted hedgehog (*Erinaceus roumanicus*) and the European hedgehog in the city of Poznań, Poland: **a** abundance of *Ixodes ricinus*, **b** prevalence of *I. ricinus*, **c** abundance of *I. hexagonus*, **d** prevalence of *I. hexagonus*, **e** abundance of *Archaeopsylla erinacei*, and **f** prevalence of *A. erinacei*. Estimates are provided separately for male and female hosts and are presented with standard errors

The abundance of *I. ricinus* was higher in *E. roumanicus* than in *E. europaeus* (species effect in Table 1; Fig. 1a) and differed among seasons, with seasonality affected by host sex and tick stage (season, season × sex, and season × stage effects in Table 1; Table 2). The abundance of *I. ricinus* was high in spring and declined throughout summer and fall, but this pattern was more pronounced in males (Fig. 1a). Males also carried more ticks than females (sex effect in Table 1). Particular tick stages differed in abundance (stage and season × stage effects in Table 1; Table 2).

**Table 1:** Factors influencing abundance and prevalence of ticks (*Ixodes ricinus* and *Ixodes hexagonus*) and fleas (*Archaeopsylla erinacei*) infesting northern white-breasted hedgehogs (*Erinaceus roumanicus*) and European hedgehogs (*Erinaceus europaeus*) in urban environment in the city of Poznań, Poland

Variable^a^	Abundance			Prevalence		
	χ*2*	df	*P*	χ^2^	df	*P*
(a) *Ixodes ricinus*						
Species	6.04	1	0.014	2.04	1	0.153
Season	79.47	2	<0.001	35.22	2	<0.001
Sex	37.06	1	<0.001	14.91	1	<0.001
Stage	110.94	2	<0.001	76.06	2	<0.001
Species × sex	–	–	–	3.69	1	0.055
Season × sex	6.89	2	0.032	–	–	–
Season × stage	59.37	4	<0.001	57.90	4	<0.001
(b) *Ixodes hexagonus*						
Species	2.05	1	0.152	1.92	1	0.166
Season	6.46	2	0.040	6.07	2	0.048
Sex	10.15	1	0.001	8.66	1	0.003
Stage	32.88	2	<0.001	24.90	2	<0.001
Season × sex	25.16	2	<0.001	24.38	2	<0.001
(c) *Archaeopsylla erinacei*						
Species	9.98	1	0.002	11.31	1	<0.001
Season	22.30	2	<0.001	22.13	2	<0.001
Sex	3.60	1	0.058	2.45	1	0.118
Species × season	5.90	2	0.052	–	–	–

^a^“Species” denotes host species (*E. roumanicus* and *E. europaeus*), “season” represents season of the year (spring, summer, autumn), “sex” represents sex of the host, and “stage” denotes tick stage (larva, nymph, female). See the “Materials and methods” section for further explanations

**Table 2:** Seasonal changes in the abundance of tick stages infesting northern white-breasted and European hedgehogs (*Erinaceus roumanicus* and *Erinaceus europaeus*, respectively) in urban environment in the city of Poznań, Poland

	*E. roumanicus*			*E. europaeus*		
	Spring	Summer	Fall	Spring	Summer	Fall
(a) *I. ricinus*						
Larvae	0.01 (0.00–0.04)	0.07 (0.03–0.19)	0.02 (0.01–0.09)	0.01 (0.00–0.02)	0.03 (0.01–0.11)	0.01 (0.00–0.04)
Nymphs	1.28 (0.71–2.29)	0.47 (0.22–0.99)	0.11 (0.05–0.26)	0.46 (0.21–0.99)	0.23 (0.09–0.59)	0.05 (0.02–0.15)
Females	0.96 (0.51–1.79)	0.13 (0.07-0.26)	0.06 (0.03–0.14)	0.47 (0.20–1.11)	0.06 (0.03–0.16)	0.03 (0.01–0.09)
(b) *I. hexagonus*						
Larvae	0.51 (0.26–1.00)	0.35 (0.18–0.66)	0.28 (0.13–0.57)	0.36 (0.16–0.83)	0.27 (0.12–0.61)	0.20 (0.08–0.47)
Nymphs	1.50 (0.85–2.64)	1.01 (0.58–1.76)	0.76 (0.39–1.46)	1.03 (0.49–2.16)	0.70 (0.32–1.54)	0.56 (0.25–1.23)
Females	0.71 (0.40–1.25)	0.49 (0.28–0.86)	0.38 (0.20–0.73)	0.49 (0.23–1.05)	0.34 (0.16–0.73)	0.28 (0.13–0.61)

Estimates were obtained with generalized linear mixed models (see the “Materials and methods” section) and are presented with 95 % confidence intervals

In contrast to its abundance, prevalence of *I. ricinus* was not affected by the host species (non-significant species effect in Table 1). However, the two hedgehog species differed in the prevalence of *I. ricinus* on males and females: *I. ricinus* prevalence was more biased toward males in *E. europaeus* than in *E. roumanicus* (sex and species × sex effects in Table 1; Fig. 1b). Similarly to the abundance, prevalence of *I. ricinus* differed among particular seasons and tick stages (season, stage, and season × stage effects in Table 1).

### I. hexagonus

In the case of *I. hexagonus*, patterns of abundance and prevalence were very similar and did not differ between *E. roumanicus* and *E. europaeus* (non-significant species effect in Table 1). Both abundance and prevalence of *I. hexagonus* were higher for male than for female hosts and differed among particular tick stages (significant sex and stage effects in Table 1; Table 2). The main effect of season on *I. hexagonus* infestation patterns was statistically significant, but weak (Table 1 and Fig. 1c, d). However, there were marked differences in seasonality of infestation between male and female hosts (Table 1): infestation of male hosts was high in spring and summer, and infestation of females was high in spring and fall (Fig. 1c, d).

### Co-infestation with *I. ricinus* and *I. hexagonus*

Simultaneous infestation with both tick species occurred more often on *E. roumanicus* than on *E. europaeus* (odds ratio χ^2^ = 4.78, df = 1, *P* = 0.029) and varied with seasons. It was highest in spring, intermediate in summer, and lowest in autumn (χ^2^ = 19.98, df = 2, *P* < 0.001). In addition, co-infestation was marginally more frequent in males than in females (χ^2^ = 3.30, df = 1, *P* = 0.069). Taking all these factors into account, the probability of co-infestation varied from 11 % (females in the fall) to 51 % (males in the spring) in *E. europaeus* and from 23 % (females in the fall) to 71 % (males in the spring) in *E. roumanicus*.

### A. erinacei

Flea abundance and prevalence were higher for *E. roumanicus* than for *E. europaeus* (species effect in Table 1c; Fig. 1e, 1f). Both abundance and prevalence of fleas differed among seasons (Table 1). In addition, the seasonal patterns of flea abundance marginally differed between the two hedgehog species (species × season interaction in Table 1), with flea abundance increasing from spring to fall in *E. roumanicus*, but not in *E. europaeus* (Fig. 1e, f). Finally, flea abundance was marginally higher in male hosts, regardless of hedgehog species (sex effect in Table 1).

## Discussion

Although northern white-breasted hedgehogs and European hedgehogs have similar biology and appear to share the same species of ectoparasites (Reeve 1994; Sommer 2007; Gaglio et al. 2010; Földvári et al. 2011; Pfäffle et al. 2014), we found that ectoparasite burdens of sympatric populations of these two species are different. *E. roumanicus* carried more *I. ricinus* ticks and *A. erinacei* fleas than *E. europaeus*. On the other hand, the two hedgehog species did not differ in *I. hexagonus* infestation parameters. These results complement a study by Pfäffle et al. (2014), who reported that *E. roumanicus* and *E. europaeus* in their contact zone in Czech Republic differed in the abundance of intestinal endoparasites.

Interspecific differences in parasite load depend on many behavioral, ecological, and physiological factors. Infestation with parasites might be influenced, e.g., by the density of host population (Anderson and May 1979; Arneberg 2001; Krasnov et al. 2002; Brunner and Ostfeld 2008), social organization (Altizer et al. 2003; Monello and Gompper 2010), variation in parasite densities within home range (Calabrese et al. 2011), host diet composition (Ezenwa 2004; Navarro-Gonzalez et al. 2011), habitat associations (Thamm et al. 2010), and differences in immunological systems (Keesing et al. 2009). However, comparative studies on these aspects of biology of *E. roumanicus* and *E. europaeus* are virtually non-existent, highlighting a gap in our knowledge. In particular, much less is known about *E. roumanicus* than *E. europaeus*. Therefore, discussion of the causes of differences in infestation patterns found in *E. roumanicus* and *E. europaeus* must be largely speculative.

*I. ricinus* tick is a generalist exophilic species, questing for its hosts on vegetation. Therefore, its off-host activity in urban environment is restricted to relatively few areas with high vegetation cover and humidity, such as parks, gardens, or cemeteries (Dautel and Kahl 1999). Thus, it is possible that the difference between *E. roumanicus* and *E. europaeus* in infestation with *I. ricinus* is related to the differential use of the urban habitat by the two host species. For example, *E. roumanicus* could have larger homer ranges or select more heavily vegetated habitats than *E. europeaus*. However, so far, there have been no studies on home range size or habitat selection in *E. roumanicus*. Moreover, the two hedgehog species differed in the abundance, but not prevalence, of *I. ricinus*. Such pattern suggests another underlying mechanism. Interspecific differences in body mass cannot explain the differences in tick abundances, because individuals of *E. europaeus* were slightly heavier, on average, than individuals of *E. roumanicus* (mean and standard deviation, 926 ± 257 vs. 722 ± 256 g); thus, we would expect an opposite pattern. Another alternative is that *E. europaeus* is more resistant to *I. ricinus* than *E. roumanicus* and therefore is able to keep infestation at lower levels (Råberg et al. 2009). Resistance to ticks is an important factor that influences the intensity of infestation (Wikel 1996), and this trait can vary greatly among different species or even breeds of animals (Fielden et al. 1992; Piper et al. 2010).

We found no differences between the two hedgehog species in the abundance and prevalence of *I. hexagonus*. In contrast to *I. ricinus*, *I. hexagonus* specializes on hedgehogs and has a nidicolous lifestyle: it inhabits nests of its host, where it reproduces, and attaches to the host when taking a blood meal. Because of this life history and frequent use of various types of nests by hedgehogs (reproductive nests, day nests, hibernacula: Reeve 1994), this parasite has nearly constant contact with its host. Infestation levels of *I. hexagonus* might be regulated by density-dependent mechanisms, such as interference competition between larvae within the nests of hosts (Pfäffle et al. 2011). Such mechanism would be largely independent of characteristics of individual host and could explain similar levels of *I. hexagonus* infestation on *E. roumanicus* and *E. europaeus*.

Co-infections with both tick species were found considerably more often in *E. roumanicus* than in *E. europaeus*. This pattern indicates that co-feeding transmission of pathogens between the two tick species might occur more often on the former species of hedgehog. Even though *I. hexagonus* does not feed on as many species as *I. ricinus*, it might be involved in the enzootic subcycle of various pathogens such as *Borrelia* spp. or *A. phagocytophilum.* These microorganisms can be acquired by *I. hexagonus*, can be passed via co-feeding transmission to *I. ricinus*, and can subsequently infect other wildlife, pets, and humans (Pfäffle et al. 2011).

We do not discuss tick stage results in this study because the two host species did not differ in this regard and such a discussion would make this report considerably longer. The analysis of *I. ricinus* and *I. hexagonus* stage dynamics can be found in Pfäffle et al. (2011) (European hedgehog) and Dziemian et al. (2014) (northern white-breasted hedgehog).

The hedgehog flea *A. erinacei* is nidicolous, similarly to *I. hexagonus* (Morris 1973). However, patterns of the flea infestation resembled rather those of the exophilic *I. ricinus*, with ectoparasite loads higher on *E. roumanicus* than on *E. europaeus.* Nevertheless, in the case of the hedgehog flea, host species strongly influenced both abundance and prevalence of the ectoparasite, and possibly also the seasonal dynamics of infestation levels. While reasons for the differences in flea loads between the two host species are not clear, they indicate that these sister hedgehog species are not as similar in their biology as previously thought (see also Bolfiková and Hulva 2012; Pfäffle et al. 2014).

As a consequence of the reported differences in ectoparasite loads, the two hedgehog species might play a different role in cycling of vector-borne diseases in urban areas. Hosts which are most heavily infested by ticks and fleas are also most likely to be infected by pathogens (Brunner and Ostfeld 2008). Moreover, such hosts are also most likely to subsequently infect numerous naïve ectoparasites. Therefore, gauging interspecific differences in ectoparasite loads is considered to be an essential component in comprehending and controlling the transmission of vector-borne zoonotic disease (Brunner and Ostfeld 2008). However, the maintenance of particular pathogens is also strongly influenced by reservoir competence of wildlife hosts (LoGiudice et al. 2003). Therefore, the reservoir status of urban-adapted animal species such as *E. roumanicus* and *E. europaeus* could provide fruitful venues of future research (see, e.g., Levin et al. 2002; Ginsberg et al. 2005; Radzijevskaja et al. 2013). This issue is particularly urgent in the light of ongoing expansion of urban and suburban areas, which become the most important sites of interactions between humans and city-adapted wildlife hosts (Pfäffle et al. 2013).
